# Emerging Zoonotic Diseases among Pastoral Communities of Caia and Búzi Districts, Sofala, Mozambique: Evidence of Antibodies against Brucella, Leptospira, Rickettsia, and Crimean-Congo Hemorrhagic Fever Virus

**DOI:** 10.3390/v15122379

**Published:** 2023-12-04

**Authors:** John Oludele, Pascoal Alho, Inocêncio Chongo, Plácida Maholela, Vlademiro Magaia, Argentina Muianga, Bibiana Melchior, Telma Isaías, Aline Gatambire, Edna Zimba, Emídio Nhavoto, Paulo Notiço, Pedro Inguana, Juma Cantoria, Virgílio António, Vanessa Monteiro, Sádia Ali, Osvaldo Inlamea, Eduardo Samo Gudo

**Affiliations:** 1 Instituto Nacional de Saúde, Marracuene 3943, Mozambique; pascoal.alho@ins.gov.mz (P.A.); inocencio.chongo@ins.gov.mz (I.C.); placida.maholela@ins.gov.mz (P.M.); argentina.muianga@ins.gov.mz (A.M.); bibiana.melchior@ins.gov.mz (B.M.); telma.isaias@ins.gov.mz (T.I.); alineconfiance@gmail.com (A.G.); edna.zimba@ins.gov.mz (E.Z.); emidiofernandonhavoto@gmail.com (E.N.); paulonotico2050@gmail.com (P.N.); pedrofelicianno@gmail.com (P.I.); jumacantoria5@gmail.com (J.C.); virgilio.antonio@ins.gov.mz (V.A.); vanessa.onofre@ins.gov.mz (V.M.); sadia.pereira@ins.gov.mz (S.A.); osvaldo.inlamea@ins.gov.mz (O.I.); eduardo.samogudo@ins.gov.mz (E.S.G.); 2 Centro de Biotecnologia, Universidade Eduardo Mondlane, Maputo CP 257, Mozambique; magaiavlade@gmail.com; 3 Center for International Health, Division of Infectious Diseases and Tropical Medicine, LMU University Hospital, Ludwig Maximilian University of Munich, 80802 München, Germany

**Keywords:** emerging zoonotic diseases, antibodies against Brucella, Leptospira, Rickettsia and CCHFV, asymptomatic pastoralists

## Abstract

Background: Emerging zoonotic diseases are an increasing threat to public health. There is little data on the seroprevalence of zoonotic diseases among pastoralists in the country. We aim to carry out a cross-sectional study on the prevalence of major zoonotic diseases among pastoral communities in the Caia and Búzi districts. Methods: Between January and December 2018, a questionnaire was used to solicit socio-demographic data from consenting pastoralists with the collection of blood samples in the Caia and Búzi districts of the Sofala province. All samples were tested using ELISA commercial reagents for the detection of IgM antibodies against Brucella and Leptospira. Likewise, IgM and IgG antibodies against Rickettsia and CCHFV were determined using ELISA kits. Results: A total of 218 samples were tested, of which 43.5% (95/218) were from the district of Caia and 56.4% (123/218) from the Búzi district. Results from both districts showed that the seroprevalence of IgM antibodies against Brucella and Leptospira was 2.7% (6/218) and 30.3% (67/218), respectively. Positivity rates for IgM and IgG anti-Rickettsia and CCHFV were 8.7% (19/218), 2.7% (6/218), 4.1% (9/218), and 0.9% (2/218), respectively. Conclusions: Results from our study showed evidence of antibodies due to exposure to Brucella, Leptospira, Rickettsia, and CCHFV with antibodies against Leptospira and Rickettsia being the most prevalent. Hence, laboratory diagnosis of zoonotic diseases is essential in the early detection of outbreaks, the identification of silent transmission, and the etiology of non-febrile illness in a pastoral community. There is a need to develop public health interventions that will reduce the risk of transmission.

## 1. Introduction

Zoonotic diseases are defined, according to the World Health Organization, (WHO) as diseases and infections that are transmitted between humans and vertebrate animals. Emerging and re-emerging zoonoses are newly appearing or have existed previously in a population but with an increasing incidence and geographic spread [1,2].

It is noteworthy that a significant proportion, up to 75%, of new or emerging infectious diseases are of zoonotic origin [3,4]. Globally, an estimated 2.5 billion cases of human illness and 2.7 million deaths are due to zoonoses annually [5]. Africa has been affected by several zoonotic diseases, ranging from endemic zoonoses, such as brucellosis and leptospirosis, to neglected zoonoses, such as rabies and onchocerciasis, to emerging zoonoses, such as anthrax, yellow fever, Ebola, Lassa fever, and COVID-19. This fact is due to several factors, such as a conducive tropical climate, an abundance of wild animals, animal husbandry including livestock transhumance, and interactions between animals and humans at various interfaces. These factors coupled with climate change provides favorable conditions for the risks of zoonotic diseases.

Brucellosis is a common zoonosis and constitutes a threat to public health in countries where control measures are not systematically applied. The disease is caused by gram-negative, facultative intracellular bacteria of the genus Brucella. There are various species, such as *B. abortus*, *B. melitensis*, and *B. suis*, found in cattle, goats, sheep, and pigs, respectively. However, the species that is most prevalent in human brucellosis is *B. melitensis* [6,7]. Brucellosis infection is through contact with infected animals, meats, aborted fetuses, carcasses, and ingestion of unpasteurized milk. In Mozambique, there are no reported data of infection in humans, however, in animals, the seroprevalence of brucellosis was detected in wildlife with 27.4% (17/62) in buffaloes, and 9.8% (13/133) in domestic animals (cattle). It is an occupational threat common to veterinarians, pastoralists, slaughterhouse workers, and laboratory personnel [8].

Leptospirosis is a zoonotic infectious disease caused by a pathogenic bacteria called Leptospira. It has a worldwide distribution but is common in tropical and subtropical regions with high rainfall [9,10]. It can be described as a disease resulting from climate change and is considered one of the main zoonoses that causes morbidity and mortality. According to Bharti et al., leptospirosis has emerged as a globally important infectious disease occurring in rural and urban environments of industrialized and developing countries. Transmission can occur when infected urine is in contact with skin cuts and abrasions and inhalation of contaminated aerosols [11,12]. Studies that were conducted in Mozambique by Ribeiro et al. (2017) and Mugabe et al. (2023) have shown leptospirosis among non-malaria febrile patients in the southern, and central regions of the country [13,14]. According to Comia et al. pathogenic species of *Leptospira* was identified in rodents using molecular methods in the northern Nampula province. In addition, factors such as rodent infestation, limited awareness of the disease, lack of access to clean water, insufficient resources for waste collection, increased crowding of households, poor sanitation environment, and deterioration of living conditions are risk factors for Leptospira infection in Mozambique [15].

Rickettsioses are zoonotic diseases caused by obligate intracellular gram-negative bacteria. In Mozambique, a study by Magaia et al. showed that Spotted Fever Group Rickettsia, represented by *R. africae*, widely circulates in Amblyomma ticks collected in the south and central regions of Mozambique [16,17,18]. In another study carried out by Ana Matsimbe et al., molecular methods were used to identify *Rickettsia africae* in Amblyomma ticks collected in cattle from the Nampula province of northern Mozambique [19].

Crimean-Congo hemorrhagic fever is a viral zoonotic disease. It is a tick-borne disease that is often found in cattle, sheep, and goats. It can be regarded as an occupational disease among pastoral communities as they are in constant contact with livestock. The primary transmission is by the bite of an infected tick Hyalomma. The pathogenic agent is a virus of the Bunyanviridae family. The secondary transmission can be through contact with infected animal blood or tissue [20,21]. The country has reported its first serologic evidence of Crimean-Congo hemorrhagic fever among febrile patients in the Caia district, Quelimane and Maputo cities [22].

The burden of zoonotic diseases may be difficult to estimate due to a lack of diagnostic capacity and under-reporting of cases. The prevalence of zoonotic diseases among pastoralists, livestock farmers, and their communities is often more complicated due to the nomadic nature of their work as they search for green pastures [23].

Notably, about 80% of the Mozambique population lives in rural areas where the main source of living is agriculture and livestock farming. The continuous contact with animals among pastoralists and their communities could serve as a means of propagating zoonotic infections in the community [24].

Data on the prevalence of zoonotic diseases among pastoral communities are scarce in Mozambique. In this research, we aim to study the occurrence of antibodies against Brucella, Leptospira, Rickettsia, and Crimean-Congo hemorrhagic fever virus (CCHFV) and estimate their prevalence among pastoralist communities of the Caia and Búzi districts.

## 2. Material and Methods

### 2.1. The Study Area and Population

**Figure 1:**
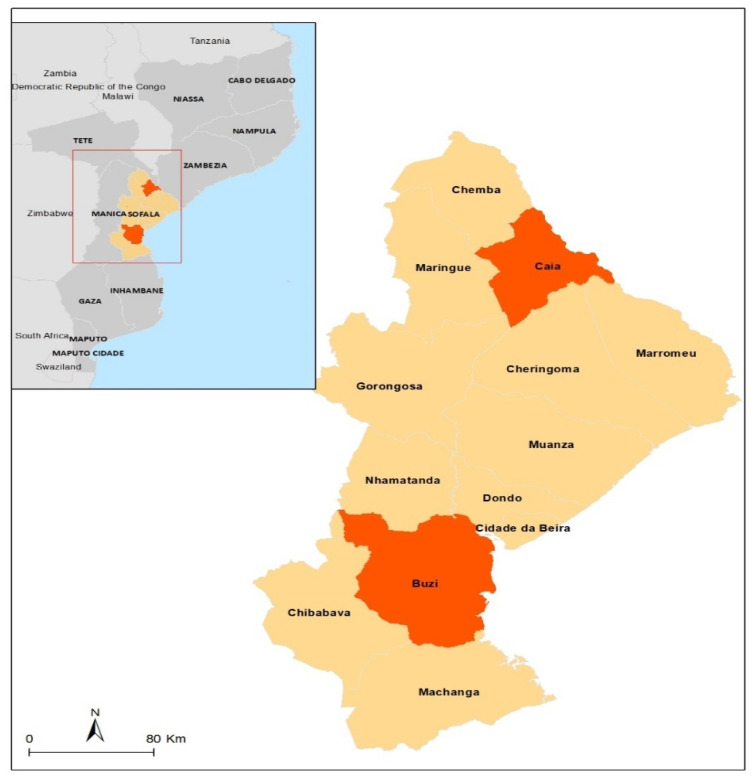
A map showing the geographic location of the Búzi and Caia districts, Sofala province.

The study was conducted in the districts of Caia and Búzi, both located in the province of Sofala (See Figure 1) during the period from January to November 2018. These districts were selected because they are rural areas with a tropical-humid climate and have large cattle, goat, sheep, and pig, rearing both on a family scale, as well as small and medium scales. The population of these districts lives essentially from the agricultural-livestock activity. Due to their location, these districts have suffered annually from the impacts of extreme weather events and exacerbating health problems. The average monthly rainfall is 77 mm, the average temperature is 26.3 °C, the relative humidity is 73.1%, and the absolute minimum and maximum temperatures of 10 °C and 42 °C, respectively [25].

### 2.2. Study Design and Sample Size Estimation

The recruitment of participants was carried out in mobile units or households. Eligibility criteria for participants in the study include, firstly, residency in the pastoral community for a minimum of three months to allow exposure to zoonotic pathogens and the development of antibodies. Secondly, given consent from adults or from guardians or parents of children between the ages of five and seventeen years. Thirdly, the participant must be apparently healthy without fever symptoms. Usual shepherd and cattle rancher concentration sites and areas around animal bath ponds and vaccination sites with the coordination of the District Service for Economic Activities of the Caia and Búzi districts (SDAE) were selected.

The sample size (n) was calculated based on the formula proposed by Daniel and Cross et al. [26] to predict a prevalence of 3% of one or more zoonosis:

n = (z^2^ × p (1 − p))/d^2^, where (z) is the statistic corresponding to the level of confidence,

(p) is the expected prevalence obtainable from the same studies, or a pilot study conducted by the researchers, and (d) is the precision that corresponds to effect size.

### 2.3. Blood Sample Collection and Laboratory Analysis

A standardized questionnaire was applied to each participant. The recruitment period from the administration of the questionnaire to the collection of the sample lasted an average of 30 min. Approximately 10 mL of whole blood samples were collected from eligible participants using the venipuncture technique by trained personnel.

Blood samples were transported from the field in a cold chain (2–8 °C) to the local health facility laboratory where they were centrifuged to obtain serum and plasma samples which eventually were transported in dry ice to the Virology Laboratory of the Instituto Nacional de Saúde, Marracuene, Maputo, and stored in freezers at −80 °C until testing. All blood samples were serologically tested using commercial ELISA kits strictly following the manufacturer’s instructions. The principles of good clinical and laboratory practice were observed in the realization of the study.

Samples were serologically tested for IgM antibodies against *Brucella* sp. using a Brucella IgM ELISA kit from Diagnostic Automation Inc. (Los Angeles, CA, USA). All reagents used for the analysis were kept at room temperature for 30 min before use. A 5 microliter (μL) of serum was added to 500 μL of sample diluent. A total of 100 μL of the diluted samples, ready-to-use standards, and controls were added to the wells of a microtiter plate coated with Brucella antigen. The plate was covered and incubated at room temperature for 1 h. The plate was emptied and 300 μL of diluted washing solution was added to wash the plate 3 times using an automated plate washer. A gentle tapping of the microtiter plate on a tissue cloth was performed to remove excess washing buffer. The 100 μL of ready-to-use conjugate was added to the wells, covered, and incubated for 30 min at room temperature. The process of washing was repeated, as mentioned above, and 100 μL of ready-to-use Tetra-methyl benzidine (TMB) substrate was added to the wells, covered, and incubated for 20 min in the dark. The 100 μL of ready-to-use stop solution was added into the wells to terminate the substrate action. The absorption reading was performed within an hour with a microplate reader at 450 nm wavelength (optionally reference wavelength at 620 nm). Samples whose optical densities were above the cutoff standard are considered positive for the presence of antibodies against Brucella, while values below the cutoff standard are negative. Indeterminate samples are repeated. According to the manufacturer’s claim, the specificity and sensitivity of the kit are said to be 100% and, in the detection of IgM antibodies against Brucella, there is no cross-reactivity between Brucella and *Bordetella pertussis*. (Diagnostic Automation, Inc. Los Angeles, CA, USA).

An SD Leptospira IgM ELISA kit was used for the detection of anti-Leptospira IgM antibodies. All reagents used for the analysis were kept at room temperature for 30 min before use. A 10 μL of serum was added to 990 μL of sample diluent. A total of 100 μL of diluted samples, positive and negative controls, were added to the wells of the coated microtiter plate and were covered with an adhesive seal, mixed well, and incubated at 37 °C for 30 min. The wells were aspirated and washed 5 times with 350 μL of diluted buffer solutions using an automated plate washer. A gentle tapping of the inverted microtiter plate on an absorbent tissue paper was performed to remove excess washing buffer. The 100 μL of diluted enzyme conjugate was added to each well, covered, and incubated for 30 min at 37 °C. The process of washing was repeated as mentioned above. The 100 μL of the mixed TMB substrates A and B solutions were added to the wells, covered, and incubated for 10 min at room temperature. A blue color developed. The 100 μL of stop solution was added to the wells. The absorption of the wells reading was performed within an hour with a bichromatic spectrophotometer at a 450 nm wavelength with the reference wavelength at 620 nm. A sample with an optical density equal to or greater than the calculated cutoff value is said to be positive for anti-Leptospira IgM, while a lower value than the cutoff is indicative of a negative result. The SD Leptospira IgM ELISA kit has a sensitivity of 97.2% in comparison with cases of antibody positivity using MAT. Negative serum specificity is 99.1%. The test kit showed no cross-reactivity with other pathogens such as Brucella, scrub typhus, toxoplasma, and chlamydia. (Standard Diagnostics, Inc. 65, Borahagal-ro, Giheung-gu, Yongin-si, Gyeonggi-do 17099. Republic of Korea).

In the diagnosis of scrub typhus (formerly Rickettsia), we used ELISA assays targeting IgM and IgG antibodies to *Orientia tsutsugamushi*. We allowed all reagents for the analysis to attain room temperature before use. A 4 μL of serum and controls were added to 396 μL of sample dilution buffer. Both positive and negative controls were assayed in duplicate. A total of 100 μL of diluted samples and controls were added to the wells of the coated microtiter ELISA plate. The plate was covered with parafilm and incubated at 37 °C in an incubator for 30 min. The plate was emptied and 300 μL of diluted wash buffer was added to wash the plate 6 times using an automated plate washer. A gentle tapping of the microtiter plate on a tissue cloth was performed to remove excess washing buffer. The 100 μL of ready-to-use enzyme–HRP conjugate was added to the wells, covered with parafilm, and incubated for 30 min at 37 °C in an incubator. The process of washing was repeated as mentioned above and 150 μL of EnWash was added to the wells and incubated for 5 min without any cover on the plate at room temperature (20–25 °C). The process of washing was repeated as mentioned above. The 100 μL of liquid TMB substrate per well was added and incubated at room temperature for 10 min in a dark place without any cover plate. The 50 μL of stop solution was added into the wells. The reading of the optical density was performed at a 450 nm wavelength with a microtiter plate reader. The test was said to be valid if the optical density (OD) of the negative control was less than 0.2 and the OD of the positive control was more than 0.5; the discrimination capacity must also be greater or equal to 5.0. Samples with absorbent values higher than the cutoff are said to be positive. (Scrub Typhus Detect, In Bios International, Inc. 307 Westlake Ave N #300, Seattle, WA 98109, USA).

Antibodies against the Crimean-Congo hemorrhagic fever virus were detected using enzyme-linked immunosorbent assays from the Vector Best commercial kit for the detection of IgM and IgG antibodies. All reagents used for the analysis were kept at 18–25 °C for 60 min before use. A 10 μL of serum was added to 90 μL of serum dilution buffer (SDB). Then, 100 μL of the diluted samples and ready-to-use controls were added to the wells of a microtiter plate coated with CCHFV antigen. The plate was covered and incubated at 37 °C for 60 min in an incubator. The plate was emptied and 400 μL of diluted washing solution was added to wash the plate 5 times using an automated plate washer. A gentle tapping of the microtiter plate on an absorbent tissue was performed to remove excess washing buffer. The 100 μL of ready-to-use conjugate was pipetted to the wells and the plate was covered and incubated for 30 min at 37 °C in an incubator. The process of washing was repeated as mentioned above and 100 μL of ready-to-use TMB solution was added to the wells, covered, and incubated at 18–25 °C for 25 min in the dark. The 100 μL of ready-to-use stop solution was added to the wells. The absorption reading was performed within an hour with a microplate reader at a 450 nm wavelength (optionally reference wavelength of 620 nm).

The cutoff was calculated by adding 0.2 to the average of the ODs of the 2 negative control wells. The tests are considered valid if the optical density of the negative control is not more than 0.25 and positive control should be or above 1.0. Sample positivity is indicated by an optical density that is greater than 1. Negative samples have an OD less than 0.8, while unequivocal results between 0.8 and 1.0 are repeated. (Vector Best, 630117, Novosibirsk, P.O.B. 492, Russia).

### 2.4. Data Entry and Statistical Analysis

With the aid of the completed questionnaires, demographic data, and results of laboratory analysis of serum samples were entered into a Microsoft Excel version 2010 database and exported to the SPSS version 20.0 statistical package. The analysis and the frequencies of occurrence of *Brucella* sp., *Leptospira* sp., Rickettsia, and CCHFV in different participants, as defined by variables such as age, gender, occupation, educational level, and place of origin, were performed using the Software R version 4.1.3. For the significance of model parameters, the Wald test was used at a 5% significance level to determine risk factors and odds ratios. In the analyses, strata were created by districts where the risk factors for each stratum were determined, and then the risk factors were determined without considering the strata. Univariate and multivariate binary logistic regression analyses were used.

## 3. Results

### 3.1. Socio-Demographic Characterization

A total of 218 individuals participated in the study, of whom 95 were from the district of Caia and 123 from the district of Búzi. The distribution of participants in relation to gender: 87.4% (83/95) were males in the Caia district and 74.7% (92/123) were males in the Búzi district. The median age of participants from the Caia district and Búzi district were 33 and 35 years, respectively. In both districts, most of the participants, 48.4% (46/95) in Caia and 45.5% (56/123) in Búzi, were in the age group between 30 and 59 years, closely followed by the age group between 15 and 29 years (see Table 1).

### 3.2. Frequency of Antibodies against Brucella, Leptospira, Rickettsia, and CCHFV in Caia and Búzi Districts

In the district of Caia, IgM antibody frequency against *Brucella* sp. and *Leptospira* sp. was 2.1% and 45.3%, respectively. The frequency of IgM and IgG antibodies against *Rickettsia* sp. and the Crimea Congo Hemorrhagic Fever Virus was 6.3%, 1.0%, 5.3%, and 1.0%, respectively. See Table 2 for the frequencies of antibodies against Brucella, Leptospira, Rickettsia, and CCHFV in both districts.

In the district of Búzi, IgM antibody frequency against *Brucella* sp. and against *Leptospira* sp. was 2.4% and 19.5%, respectively. The frequency of IgM and IgG antibodies against *Rickettsia* sp. and the Crimea Congo Hemorrhagic Fever Virus was 13.8%, 4.0%, 3.3%, and 0.8%.

## 4. Discussion

### 4.1. Seroprevalence of Antibodies against Brucella

In a study performed by Mendonça et al. (2015) in two slaughterhouses of Maputo city and province, the overall seroprevalence of brucellosis in slaughtered animals was 16.5% 56/340 [27]. The improper handling of aborted fetuses, drinking of unpasteurized milk, and eating of raw meat were part of the risk factors involved in the transmission of brucellosis from cattle to man [28]. In this study, although there is no significant relationship between the variables such as occupation, gender, and age, the seroprevalence of brucellosis found among cattle owners and herders is 2.4% as recorded in the Búzi district and occurs only in males (see Appendix A, Table A1). A similar result of 3.0% prevalence was obtained among inhabitants of pastoral areas in Eritrea, while a higher prevalence of 4.5% and 7.1% was observed among veterinarians and dairy farm workers, respectively, according to Schelling et al. (2003). Our results show a lower prevalence in comparison to a study on the evaluation of the seroprevalence of brucellosis in humans that was conducted among non-febrile participants of three nomadic communities of Chad with a prevalence of 3.8%, according to Schelling et al. (2003) [29,30]. Similar results were reported among hospitalized febrile patients in northern Tanzania where the seroprevalence of Brucellosis was 3.5% according to Bouley et al. (2012) [31]. On the other hand, a higher prevalence of 4.3% was observed among febrile patients in contact with animals, as shown in the study conducted in southwestern Uganda by Migisha et al. (2012) [32].

### 4.2. Seroprevalence of Antibodies against Leptospira

The samples for the study were collected during the rainy season with high humidity and heavy rainfall, which creates an enabling environment for the propagation, survival, and maintenance of *Leptospira* sp. in circulation. Although the district of Caia has a comparatively smaller sample size, it has the highest level of Leptospira seroprevalence of 45% (see Appendix A, Table A2) The age groups most affected are between 15 and 29 and 30 and 59 years and occurred in both male and female participants with a higher percentage of positivity among males, even though there is no significant relationship between gender, age, and occupational groups such as cattle owners, herders, and domestic workers. The above age and professional groups are probably exposed frequently to rodents, animal feces, and urine such that a high prevalence of leptospirosis may indicate an ongoing exposure to the bacteria. A somewhat high Leptospira seroprevalence of 35% was found by Dreyfous et al. in a study among hospital patients in a rural western Uganda district of Hoima [33]. The agro pastoralist communities in the Katavi, Tanzania has the overall sero-prevalence of 26% (244/952). This is lower than the one obtained in this study. However, in an urban setting, a much lower Leptospiral prevalence among febrile patients in Maputo was found to be 8.8% of 160 patients [34,35].

### 4.3. Seroprevalence of Antibodies against Rickettsia

There is both serological and molecular evidence of scrub typhus in Africa, although most of the cases have been reported in the Asia Pacific region, central Asia, and the Middle East [36,37]. As stated earlier, Magaia et al. [18] identified *Rickettsia africae* in Amblyomma ticks in southern and central Mozambique. In this study, the highest seroprevalence of TG Rickettsia is (13.4%) (see Appendix A, Table A3) found in the Búzi district mostly among cattle herders and owners and is probably due to greater exposure to cattle. The frequency of the antibodies observed did not show a statistically significant relationship with the gender, age, and profession of the participants. The observed prevalence was lower in comparison to the high seroprevalence of TG Rickettsia recorded in southwestern Tanzania, with a site-specific seropositivity of 17.8% [38]. Cross-reactivity of *Orientia* with other Rickettsiae is said to be rare. A study on Rickettsia carried out in Angola among febrile patients who have contact with domestic animals shows a low seroprevalence, where 3 patients were positive among 87 [39].

### 4.4. Seroprevalence of Antibodies against CCHFV

According to Muianga et al., [22] a prevalence of 2.7% was observed against CCHFV IgG antibodies among febrile patients in Maputo, Caia, and Quelimane. The seroprevalence of IgM and IgG antibodies against CCHFV in the Caia and Búzi districts are 5.3%, 1.3%, 3.1%, and 1.3%, respectively. We observed that all cases of positivity were found among only male participants and the professional group affected included cattle owners, herders, and abattoir workers, yet there is no significant relationship between these variables and the observed frequencies of antibodies against CCHFV (see Appendix A, Table A4); this could be because more males are involved in cattle rearing. In the Sangailu and Ijara districts of northern Kenya, according to Lwande et al., a higher prevalence of 23% and 14%, respectively, was reported among the febrile nomadic pastoralist community. As expected, the seroprevalence of antibodies against CCHFV was higher in febrile pastoralists than in our results among asymptomatic pastoralists [40]. 

**Figure A1:**
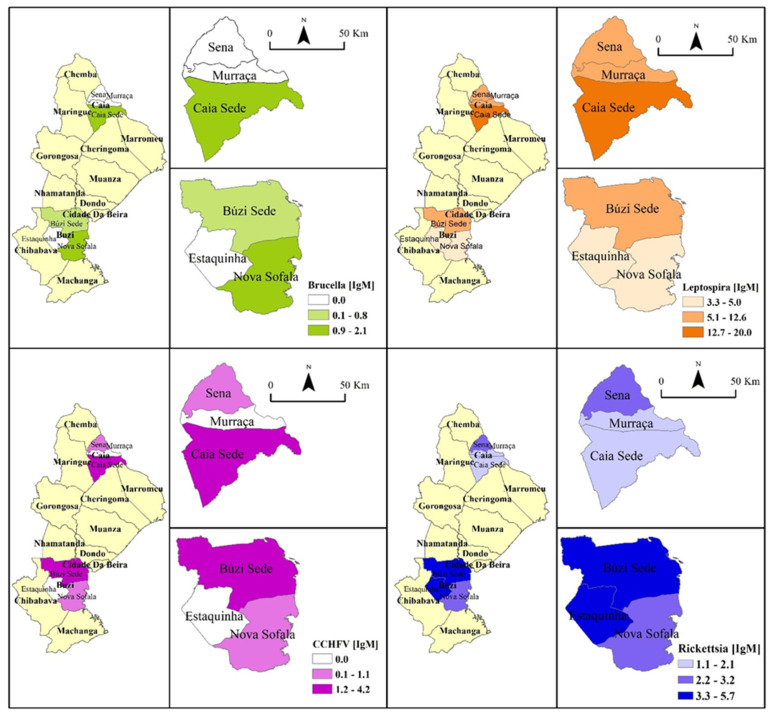
Occurrence of zoonotic diseases in the Búzi and Caia districts.

Figure A1 showed the relative distribution of IgM antibodies against Brucella, Leptospira, Rickettsia, and Crimean-Congo Hemorrhagic Fever virus in the areas understudy.

We acknowledged a study limitation because of the lack of IgM results for Brucella and Leptospira and the lack of confirmatory assays. We consider this an exploratory study since little data have been available in recent years, and no data was previously collected from pastoralists in Mozambique. However, they are of utmost importance to understand exposure to these pathogens in the changing context of climate globally.

## 5. Conclusions

Our study showed evidence of the circulation of antibodies against Brucella, Leptospira, Rickettsia, and CCHFV among pastoralists, suggesting exposure to these emerging zoonotic pathogens. Antibodies against Leptospira and Rickettsia were the most prevalent in the two districts’ understudy, suggesting a silent and ongoing exposure and infection. Hence, laboratory analysis of zoonotic diseases should be considered, not only in febrile patients but also in healthy pastoralists, veterinarians, and slaughterhouse workers as they are in constant contact with animals. To the best of our knowledge, this is the first seroepidemiology study on emerging zoonosis among pastoralists.

## Figures and Tables

**Table 1:** Socio-demographic characteristics of the study participants in the Búzi and Caia districts.

Variables		Búzi District (*N* = 123)		Caia District (*N* = 95)	
		Frequency	%	Frequency	%
Gender	Female	31	25.2	12	12.6
	Male	92	74.7	83	87.3
Age	5–14 years	7	5.6	2	2.1
	15–29 years	49	39.8	41	43.1
	30–59 years	56	45.5	46	48.4
	≥60 years	11	8.9	6	6.3
Education status	None	18	14.6	9	9.4
	Primary	72	58.5	56	58.9
	Basic	19	15.4	19	20
	Medium	12	9.7	9	9.4
	Professional technician	2	1.6	1	1
	Higher	0	0.0	1	1
Occupation	Domestic worker	11	8.9	5.0	5.2
	Student	14	11.3	4.0	4.2
	Education professional	1	0.8	4.0	4.2
	Health professional	5	4.0	0.0	0.0
	Veterinarian	1	0.8	2.0	2.1
	Slaughterhouse worker	0	0.0	5.0	5.2
	Cattle owner	37	30.0	45.0	47.3
	Cattle herder	43	34.9	25.0	26.3
	Others	10	8.1	5.0	5.3
	None	1	0.8	0.0	0.0

**Table 2:** Frequency of antibodies against Brucella, Leptospira, Rickettsia, and CCHFV.

	Búzi (*n* = 123)		Caia (*n* = 95)	
	*N*	%	*N*	%
Brucellosis	3	2.4	2	2.1
Leptospirosis	24	19.5	43	45.3
Rickettsiosis	17	13.8	6	6.3
Crimean-Congo Hemorrhagic Fever	4	3.3	5	5.3

## Data Availability

Any supplementary data not published within the context of this article will be provided upon request by corresponding author.

## Appendix A

**Table A1:** Sociodemographic data and frequency of anti-Brucella antibodies.

		Búzi District (*n* = 123)				Caia District (*n* = 95)			
	Frequency		OR	IC	*p*-Value	Frequency	OR	IC	*p*-Value
Gender	Female	0	1.00	-	Ref	1 (1.1%)	1.00	-	Ref
	Male	3 (2.4%)	>100	-	0.996	1 (1.1%)	0.11	0.00; 3.07	0.138
Age group	5–14 years	0	1.00	0.00; Inf	1.000	0	1.00	0.00; Inf	1.000
	15–29 years	2 (1.6%)	>100	-	0.996	1 (1.1%)	>100	-	0.997
	30–59 years	1 (0.8%)	>100	-	0.997	1 (1.1%)	>100	-	0.997
	≥60 years	0	1.00	-	Ref	0	1.00	-	Ref
Education	None	0	0.00	-	0.997	1 (1.1%)	2.83	0.10; 79.7	0.485
	Primary	2 (1.6%)	0.65	0.06; 14.6	0.735	1 (1.1%)	0.00	-	0.997
	Basic	1 (0.8%)	1.00	-	Ref	0	1.00	-	Ref
	Medium	0	0.00	-	0.997	0	0.00	-	0.999
	Professional technician	0	0.00	-	0.999	0	0.00	-	0.999
	Higher	0	-	-	-	0	0.00	-	0.999
Occupation	Domestic worker	0	1.00	0.00; Inf	1.000	0	0.00	-	0.999
	Student	0	1.00	0.00; Inf	1.000	1 (1.1%)	12.00	0.41; 364	0.106
	Education professional	0	1.00	0.00; Inf	1.000	0	0.00	-	0.999
	Health professional	0	1.00	0.00; Inf	1.000	0	0.00	-	-
	Veterinarian	0	1.00	0.00; Inf	1.000	0	0.00	-	0.999
	Slaughterhouse worker	0	-	-	-	0	0.00	-	0.999
	Cattle owner	0	1.00	-	Ref	1 (1.1%)	1.00	-	Ref
	Cattle herder	3 (2.4%)	1.00	0.00; Inf	0.997	0	0.00	-	0.997
	Others	0	1.00	0.00; Inf	1.000	0	0.00	-	0.998
	None	0	1.00	0.00; Inf	1.000	0	-	-	-

Ref indicates the variable used as a reference for comparison in the category and Inf means infinite.

**Table A2:** Sociodemographic data and frequency of anti-Leptospira antibodies.

		Búzi District (*n* = 123)				Caia District (*n* = 95)			
	Frequency		OR	IC	*p*-Value	Frequency	OR	IC	*p*-Value
Gender	Female	4 (3.3%)	1.00	-	Ref	5 (5.3%)	1.00	-	Ref
	Male	20 (16.3%)	1.87	0.64; 6.89	0.289	38 (40.0%)	1.18	0.35; 4.28	0.789
Age group	5–14 years	0	0.00	-	0.991	0	0.00	-	0.988
	15–29 years	8 (6.5%)	0.52	-	0.402	19 (20.0%)	0.86	0.14; 5.15	0.867
	30–59 years	13 (10.6%)	0.81	-	0.773	21 (22.1%)	0.84	0.14; 4.96	0.841
	≥60 years	3 (2.4%)	1.00	-	Ref	3 (3.2%)	1.00	-	Ref
Education	None	4 (3.3%)	1.52	0.29; 8.88	0.619	6 (6.3%)	5.60	1.07; 35.9	0.049
	Primary	17 (13.8%)	1.65	0.48; 7.69	0.467	5 (5.3%)	2.26	0.75; 7.77	0.165
	Basic	3 (2.4%)	1.00	-	Ref	25 (26.3%)	1.00	-	Ref
	Medium	0	0.00	-	0.989	6 (6.3%)	5.60	1.07; 35.9	0.049
	Professional techician	0	0.00	-	0.996	0	0.00	-	0.992
	Higher	0	-	-	-	1 (1.1%)	>100	-	0.991
Occupation	Domestic worker	3 (2.4%)	2.40	0.42; 12.1	0.291	1 (1.1%)	0.34	0.02; 2.55	0.354
	Students	1 (0.8%)	0.49	0.02; 3.46	0.535	3 (3.2%)	4.11	0.48; 86.6	0.237
	Education professional	0	0.00	-	0.995	2 (2.1%)	1.37	0.15; 12.3	0.764
	Health professional	1 (0.8%)	1.60	0.07; 14.0	0.699	0	-	-	-
	Veterinarian	1 (0.8%)	>100	-	0.993	1 (1.1%)	1.37	0.05; 36.1	0.828
	Slaughterhouse worker	0	-	-	-	2 (2.1%)	0.91	0.11; 6.03	0.924
	Cattle owner	5 (4.1%)	1.00	-	Ref	19 (20.0%)	1.00	-	Ref
	Cattle herder	12 (9.8%)	2.48	0.82; 8.55	0.123	13 (13.7%)	1.48	0.55; 4.01	0.432
	Others	1 (0.8%)	0.71	0.03; 5.21	0.768	2 (2.1%)	0.91	0.11; 6.03	0.924
	None	0	0.00	-	0.995	0	-	-	-

Ref indicates the variable used as a reference for comparison in the category and Inf means infinite.

**Table A3:** Sociodemographic data and frequency of anti-Rickettsia antibodies.

		Búzi District (*n* = 123)				Caia District (*n* = 95)			
	Frequency		OR	IC	*p*-Value	Frequency	OR	IC	*p*-Value
Gender	Female	4 (3.3%)	1.00	-	Ref	1 (1.1%)	1.00	-	Ref
	Male	13 (10.6%)	1.11	0.36; 4.20	0.995	5 (5.3%)	0.71	0.10; 14.2	0.864
Age group	5–14 years	0	1.00	0.00; Inf	1.000	0	0.00	-	0.995
	15–29 years	9 (7.3%)	>100	-	0.993	3 (3.2%)	0.39	0.04; 8.82	0.457
	30–59 years	8 (6.5%)	>100	-	0.993	2 (2.1%)	0.23	0.02; 5.39	0.259
	≥60 years	0	1.00	-	Ref	1 (1.1%)	1.00	-	Ref
Education	None	2 (1.6%)	1.06	0.12; 9.73	0.954	3 (3.2%)	9.00	0.95; 203	0.078
	Primary	10 (8.1%)	1.37	0.32; 9.47	0.701	1 (1.1%)	0.67	0.06; 14.8	0.747
	Basic	2 (1.6%)	-	-	Ref	2 (2.1%)	1.00	-	Ref
	Medium	2 (1.6%)	1.70	0.18; 16.1	0.622	0	0.00	-	0.994
	Professional techician	1 (0.8%)	8.50	0.27; 290	0.181	0	0.00	-	0.998
	Higher	0	-	-	-	0	-	-	0.998
Occupation	Domestic worker	0	0.00	-	0.993	0	0.00	-	0.997
	Student	2 (1.6%)	1.38	0.17; 8.04	0.732	0	0.00	-	0.997
	Education professional	1 (0.8%)	-	-	0.997	0	0.00	-	0.997
	Health professional	2 (1.6%)	5.00	0.59; 45.2	0.106	0	-	-	-
	Veterinarian	0	0.00	-	0.998	0	0.00	-	0.9983
	Slaughterhouse worker	0	-	-	-	0	0.00	-	0.997
	Cattle owner	4 (3.3%)	1.00	-	Ref	2 (2.1%)	1.00	-	Ref
	Cattle herder	7 (5.7%)	1.60	0.44; 6.59	0.482	2 (2.1%)	1.87	0.21; 16.4	0.544
	Others	1 (0.8%)	0.92	0.04; 7.23	0.941	2 (2.1%)	14.3	1.36; 165	0.022
	None	0	0.00	-	0.998	0	-	-	-

Ref indicates the variable used as a reference for comparison in the category and Inf means infinite.

**Table A4:** Sociodemographic data and frequency of anti-CCHFV antibodies.

		Búzi District (*n* = 123)				Caia District (*n* = 95)			
	Frequency		OR	IC	*p*-Value	Frequency	OR	IC	*p*-Value
Gender	Female	0	1.00	-	Ref	0	1.00	-	Ref
	Male	4 (3.3%)	>100	-	0.993	5 (5.3%)	>100	-	0.993
Age group	5–14 years	1 (0.8%)	1.67	0.06; 47.7	0.734	1 (1.1%)	>100	-	0.994
	15–29 years	2 (1.6%)	0.49	0.04; 11.1	0.573	1 (1.1%)	>100	-	0.996
	30–59 years	0	0.00	-	0.994	3 (3.2%)	>100	-	0.995
	≥60 years	1 (0.8%)	1.00	-	Ref	0	1.00	-	Ref
Education	None	1 (0.8%)	>100	-	0.997	0	1.00	-	1.000
	Primary	3 (2.4%)	>100	-	0.997	0	>100	-	0.996
	Basic	0	-	-	Ref	5 (5.3%)	-	-	Ref
	Medium	0	1.00	-	1.000	0	1.00	-	1.000
	Professional technician	0	1.00	0.00; Inf	1.000	0	1.00	-	1.000
	Higher	0	-	-	-	0	1.00	-	1.000
Occupation	Domestic worker	0	0.00	-	0.997	0	0.00	-	0.997
	Student	0	0.00	-	0.997	0	0.00	-	0.998
	Education professional	0	0.00	-	0.999	0	0.00	-	0.998
	Health Professional	0	0.00	-	0.998	0	0.00	-	-
	Veterinarian	0	0.00	-	0.999	0	0.00	-	0.998
	Slaughterhouse worker	0	-	-	-	1 (1.1%)	5.38	0.22; 70.4	0.200
	Cattle owner	1 (0.8%)	-	-	Ref	2 (2.1%)	-	-	Ref
	Cattle herder	3 (2.4%)	2.58	0.31; 53.7	0.421	2 (2.1%)	1.95	0.22; 17.2	0.517
	Others	0	0.00	-	0.997	0	0.00	-	0.997
	None	0	0.00	-	0.999	0	-	-	-

Ref indicates the variable used as a reference for comparison in the category and Inf means infinite.
